# Modified Halsted's operation for inguinal hernia repair: A new technique

**DOI:** 10.1016/j.amsu.2021.102968

**Published:** 2021-10-19

**Authors:** Omar Salem Khattab Alomar

**Affiliations:** Department of General Surgery, College of Medicine, University of Baghdad, Baghdad, Iraq

**Keywords:** Inguinal hernia, Modified Halsted's technique, Recurrence, Complications, Hernioplasty

## Abstract

**Background:**

Inguinal hernia is a frequent problem presented to surgical clinic in Iraq. Surgical treatment options for inguinal hernia are numerous, selecting the appropriate method or technique depends on different factors.

**Aim of study:**

To find a new technique for open inguinal hernia repair with no recurrence even in recurrent cases, without major complications during or after surgery, and can be used even by new surgeons with little experience.

**Patients & methods:**

A retrospective cross sectional study conducted in the Medical City teaching hospital and Private Hospitals in Baghdad, during the period from January 1, 2000, to December 30, 2016 on convenient sample of 408 Iraqi patients with inguinal hernia. The patients were treated by modified Halsted's technique by open surgery applying polypropylene mesh. The patients were followed up through frequent visits and phone calls to assess the recurrence and complications.

**Results:**

No recurrence of inguinal hernia was reported after 5–10 years follow up, while 94.9% of patients reported postoperative complications commonly oedema of spermatic cord. There was a highly significant association between male gender hernia patients and post Modified Halsted operation complications (p < 0.001). A highly significant association was observed between direct hernia and post Modified Halsted operation complications (p < 0.001). The significant risk factors related to complications were anemia and collagen disease.

**Conclusions:**

The modified Halsted's operation for inguinal hernia repair is effective in treatment of inguinal hernia with low recurrence and complications rate.

## Highlights

• Inguinal hernia repair is a common surgical operation, and there is always a search for a technique with no recurrence.
• Inguinal hernia recurrence is frequent whatever the surgical technique used; still there is an indication for open repair
• In this study we presents a new technique for open inguinal hernia repair with long follow up and no recurrence.

## 1 Introduction

Inguinal hernia is a common surgical disease all over the world with lifetime risk of 27% among men and of 3% among women [1,2]. It is defined as bulging of abdominal contents to the inguinal area [3]. The pathogenesis of inguinal hernia in adults is related mainly to disordered extracellular matrix. Abnormalities of matrix metalloproteases or inhibitors and metabolism of collagen are shown to have a major role in etiology [4]. The common risk factors for inguinal hernia are chronic obstructive pulmonary diseases, cigarette smoking, collagen diseases and underweight [5]. Inguinal hernias are classified intraoperatively into indirect, direct, scrotal, and others [6]. Diagnosis of inguinal hernia depends commonly on history and physical examination; ultrasound examination is also helpful in diagnosis [4].

The inguinal hernia surgical repair represents the treatment of choice for inguinal hernia and it is a common surgical operation done globally with more than 800,000 inguinal hernia repairs implemented annually [7]. Hernia repair aimed to alleviate symptoms, improving quality of life and preventing inguinal hernia adverse events like incarceration, obstruction and strangulation, in addition to lower rate of post-repair complications [8]. Surgical repair is the definitive treatment of inguinal hernia, which is done through open approach (tissue repair or prosthetic repair) or laparoscopic repair (Transabdominal preperitoneal procedure or Total extraperitoneal procedure) [9]. The laparoscopic surgery is mainly implemented by posterior approach and applying mesh, while the open surgical repair is based on suturing by classical anterior approach. Common suturing techniques are Bassini, Shouldice, Desarda and Lichtenstein techniques [10]. It was shown that use of surgical open mesh-based repair is accompanied with decreasing the risk of hernia recurrence in range between 50 and 75% [10,11].

Edoardo Bassini inguinal hernia repair technique was firstly described at 1887 and aimed to reconstruct the anatomy of inguinal canal. The Halsted's method in inguinal hernia repair was developed by William Steward Halsted at 1889. Difference of Halsted's method from classical Bassini method was the subcutaneous positioning of spermatic cord. Halsted left the spermatic cord at subcutaneous position after excision of most of its veins, and closure of the structures of the abdominal wall in one layer with interrupted mattress sutures. The first trail of Halsted's method which done by Halsted himself was showed one case with complication of urinary fistula caused by deep transfixing sutures [12]. Although the urinary fistula was closed spontaneously, this event revealed high risk of deep suturing in transversalis fascia and preperitoneum [13]. In next years, many surgeons developed modifications for Halsted's method like Paolo Postempski [14]. With advances of many prosthetic materials, the need for surgical repair with suturing was mainly decreased. However, high proportion of surgeons chooses the classical surgical repair methods (Bassini, Halsted's, Postempski and Shouldice surgical methods) as the treatment option for inguinal hernia till now [15]. Many international guidelines recommended surgeons selecting the best techniques in surgical treatment of inguinal hernia according to experience, available resources and risk factors related to hernia and patients [16]. Generally, the inguinal hernia post-operative surgical complications are infection, dehiscence of fascia, recurrences, neuralgia, visceral injury and mesh erosions or migration. Postoperatively, the pain may persist for six months duration [17].

Inguinal hernias are common surgical disorder presenting daily to surgical clinics in Iraq [18]. In spite of great advances of laparoscopic surgical intervention of inguinal hernia in Iraq nowadays, the classical surgical repair by open surgery remain the suitable choice due to economic reasons, low national health infrastructure and lack of well developed laparoscopic techniques [19]. For these reasons, this study aimed to find a new technique for open inguinal hernia repair with no recurrence even in recurrent cases, without major complications during or after surgery, and can be used even by new surgeons with little experience*.*

## 2 Patients and methods

This study was a retrospective cross sectional study conducted in the Medical City teaching hospital and Private Hospitals in Baghdad, during the period from January 1, 2000, to December 30, 2016. The study population was all patients with inguinal hernia admitted to hospital during study duration. Inclusion criteria were Iraqi patients from different provinces of Iraq, 18 years age and more, primary or recurrent inguinal hernias including 2nd and 3rd times recurrence. Exclusion criteria were younger age, lost to follow up and refused to participate. Ethical considerations were obtained according to Helsinki Declaration. Informed written consent was obtained after explaining the nature of the operation and its risks. The approval of ethics committee was obtained from Health Ethics Committee in college of medicine, university of Baghdad with a registration number: 149. The methods of this article were prepared according to STROCSS criteria [20]. The research was registered at research registry: https://www.researchregistry.com. With unique identifying number researchregistry 7177. A convenient sample of 408 Iraqi patients with inguinal hernia was selected after eligibility to inclusion and exclusion criteria.

Three hundred and fifty (85.78%) patients received general anaesthesia, 50 (12.25%) received spinal anaesthesia, 8 (1.96%) received local anaesthesia. The usual oblique inguinal skin incision was used, extends from just above pubic tubercle, and 1 cm above and parallel to the line of inguinal ligament. The external oblique aponeurosis (EOA) is divided from the external ring till the level of the internal ring, care being taken to avoid injury to the ilio-inguinal nerve.

The spermatic cord along with the cremasteric muscle is separated from the inguinal floor. Excision of the sac is done in all cases except in small direct hernias where it is inverted. Prolapsed preperitoneal fat "lipomas" was excised.

**Fig. 1:**
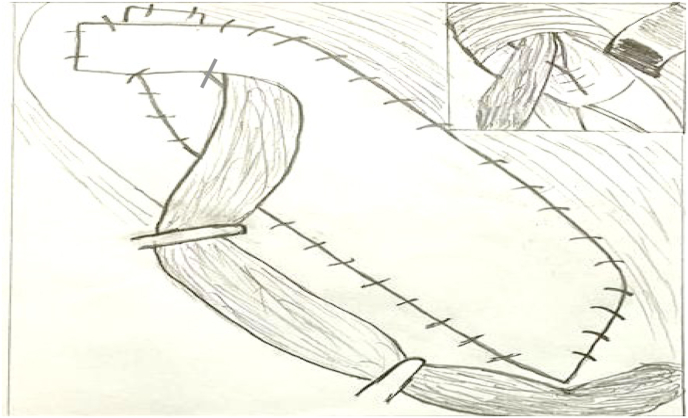
First step is narrowing of the internal ring, second step applying polypropylene mesh.

**Fig. 2:**
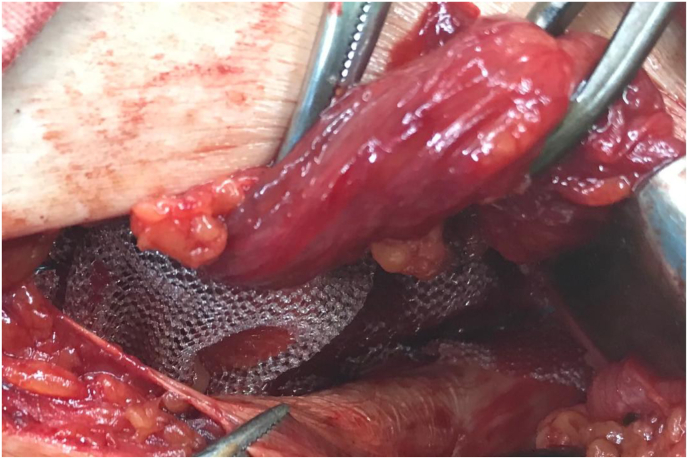
Second step, applying polypropylene mesh sutured continuously above to the conjoined tendon, and below to the reflection of the inguinal ligment making a hole in its center to allow the passage of the spermatic cord.

**Fig. 3:**
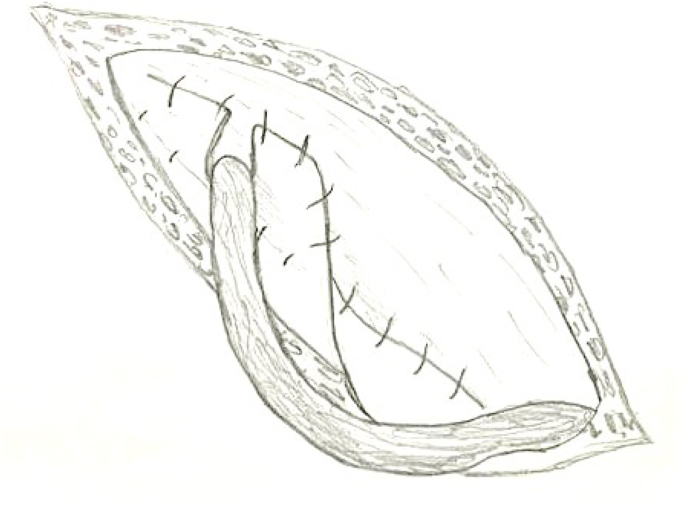
Third step, doing double breast suturing of the EOA, the medial leaf is sutured to the inguinal ligament, and the lateral leaf is sutured to overlap the medial leaf behind the cord, The cord is made to lie subcutaneously.

**Fig. 4:**
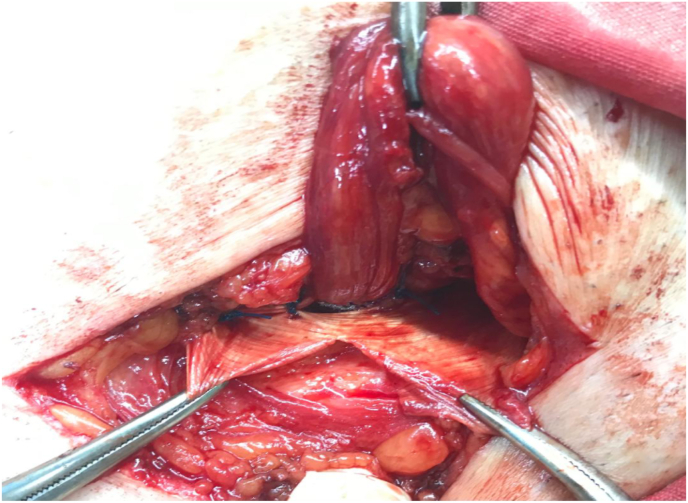
Third step, the cord is made to lie subcutaneously by bringing it through a slit in the lower flap of the EOA without constricting it.

**Fig. 5:**
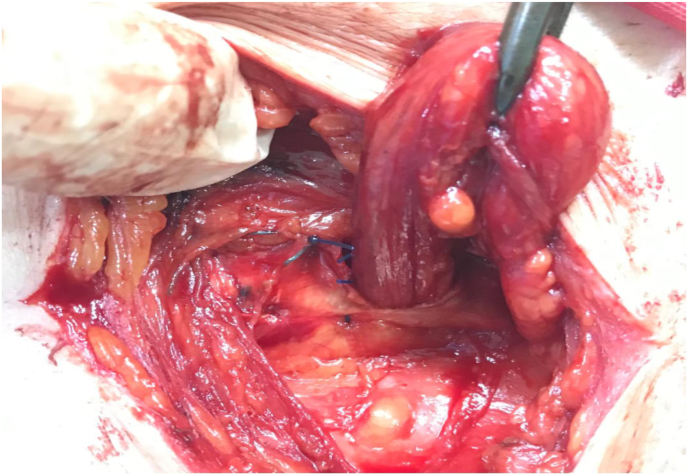
Third step, the lateral leaf is sutured to overlap the medial leaf behind the cord; the cord is made to lie subcutaneously.

Our technique consist of three steps; first step is narrowing of the internal ring if its more than 1 cm, by using 2–3 Prolene stitches number 0 after doing herniotomy. Second step is hernioplasty, to strengthen the posterior wall of the inguinal canal by applying polypropylene mesh sutured continuously above to the conjoined tendon, and below to the reflection of the inguinal ligment making a hole in its center to allow the passage of the spermatic cord, using Prolene suture number 0 (Fig. 1, Fig. 2). Third step is obliteration of the inguinal canal by doing double breast suturing of the EOA, the medial leaf is sutured to the inguinal ligament from the pubic tubercle to the level of 1–2 cm beyond the internal ring using number 1 Prolene interrupted stitches, the lateral leaf is sutured to overlap the medial leaf behind the cord, by doing double breasting to form a new posterior wall. The cord is made to lie subcutaneously by bringing it through a slit in the lower flap of the EOA without constricting it, to prevent occurrence of varicocele (Fig. 3, Fig. 4, Fig. 5). So it works as a shield to prevent recurrence, that the external oblique muscle gives additional strength to the weakened internal oblique and transverse abdominis muscle.

Ambulation was permitted from the day of surgery and normal activity was permitted after a week. All patients were discharged from hospital after 6 h. The skin subcuticular suture was removed on the 8th day. Ciprofloxacin 500 mg tablets bid, and diclofanac 50 mg tablets bid, were given for a week as prophylaxis. Follow up of patients was done after 1, 3 months and then every year, by outpatient visit, or mobile call, for assessing the early postoperative complications such as Odema of the cord, wound seroma, wound hematoma, scrotal hematoma, wound infection, and late complication like recurrence, chronic pain, testicular atrophy, and varicocele.

The patients were followed up by the researcher through frequent visits and phone calling to assess the recurrence and complications.

The data collected were analyzed statistically by Statistical Package of Social Sciences software version 22. The Chi square and Fishers' exact tests were for analysis of categorical variables accordingly. Level of significance (p value) was regarded statistically significant if it was 0.05 or less.

## 3 Results

This study included 408 patients with inguinal hernia undergone Modified Halsted operation for inguinal hernia repair with mean age of (40.2 years) and range of 18–90 years; 37.5% of patients were in age group <30 years, 18.1% of them were in age group 30–39 years, 13.7% of them were in age group 40–49 years, 12% of them were in age group 50–59 years, 10% of them were in age group 60–69 years and 8.6% of them were in age group of 70 years and more. Male inguinal hernia patients were more than females with male to female ratio of 18.4:1. The inguinal hernia sides of patients were right (52.2%), left (44.9%) and bilateral (2.9%). The inguinal hernia types of patients were commonly indirect (77.2%), followed by direct (20.8%), and direct and indirect (2%). Previous inguinal hernia recurrence was observed in 9.8% of studied patients; 75% of them had one time recurrence, 17.5% of them had two times recurrence and 7.5% of them had three times recurrence. About two thirds (61.8%) of inguinal hernia patients were followed for ten years post Modified Halsted operation, while 38.2% of them were followed for five years after the operation. Hernia recurrence post Modified Halsted operation was absent in all studied patients (Table 1).

**Table 1:** General characteristics of inguinal hernia patients.

Variable	No.	%
**Age** mean ± SD (40.2 ± 18.8 years)		
<30 years	153	37.5
30–39 years	74	18.1
40–49 years	56	13.7
50–59 years	49	12.0
60–69 years	41	10.0
≥70 years	35	8.6
**Gender**		
Male	387	94.9
Female	21	5.1
**Hernia side**		
Right	213	52.2
Left	183	44.9
Bilateral	12	2.9
**Hernia type**		
Direct	85	20.8
Indirect	315	77.2
Direct and indirect	8	2.0
**Previous recurrence**		
Yes	40	9.8
No	368	91.2
**Previous recurrence times**		
One time	30	75.0
Two times	7	17.5
Three times	3	7.5
**Follow up duration**		
Five years	156	38.2
Ten years	252	61.8
**Current recurrence**		
Yes	0	–
No	408	100.0
**Total**	**408**	**100.0**

The post Modified Halsted operation for inguinal hernia repair complications was observed in 94.9% of hernia patients, commonly oedema of cord (97.4%), followed by oedema of cord & pain on exertion (1.3%), oedema of cord & wound seroma (0.5%), oedema of cord & wound hematoma (0.5%) and oedema of cord & chronic pain (0.3%) (Table 2).

No significant differences were observed between inguinal hernia patients with postoperative complications and inguinal hernia patients without postoperative complications regarding age (p = 0.6), hernia side (p = 0.3), previous recurrence (p = 0.1).

and follow up duration (p = 0.3). There was a highly significant association between male gender hernia patients and post Modified Halsted operation complications (p < 0.001). A highly significant association was observed between direct hernia type and post Modified Halsted operation complications (p < 0.001) (Table 4).

**Table 2:** Postoperative complications of Modified Halsted inguinal hernia repair.

Variable	No.	%
Postoperative complications		
Yes	387	94.9
No	21	5.1
**Complications types**		
Oedema of cord	377	97.4
Oedema of cord & wound seroma	2	0.5
Oedema of cord & wound hematoma	2	0.5
Oedema of cord & pain on exertion	5	1.3
Oedema of cord & chronic pain	1	0.3
**Total**	**408**	**100.0**

The main risk factors for inguinal hernia observed in studied patients were smoking (31.9%), diabetes mellitus (19.6%), chronic cough (12.3%), obesity (9.8%), chronic lung disease (6.4%), anemia (5.9%), immunosuppressant (5.9%), chronic bronchitis (4.7%) and collagen disease (1.7%) (Table 3).

**Table 3:** Risk factors of inguinal hernia.

Variable	No.	%
Smoker		
Yes	130	31.9
No	278	68.1
**DM**		
Yes	80	19.6
No	328	80.4
**Obese**		
Yes	40	9.8
No	368	90.2
**Anemia**		
Yes	24	5.9
No	384	94.1
**Chronic lung disease**		
Yes	26	6.4
No	382	93.6
**Chronic bronchitis**		
Yes	19	4.7
No	389	95.3
**Chronic cough**		
Yes	50	12.3
No	358	87.7
**Collagen disease**		
Yes	7	1.7
No	401	98.3
**Immunosuppressant**		
Yes	24	5.9
No	384	94.1
**Total**	**408**	**100.0**

**Table 4:** Distribution of inguinal hernia patients' general characteristics according to postoperative complications.

Variable	Complications				P
	Yes		No		
	**No.**	**%**	**No.**	**%**	
**Age**					0.6 ^NS^
<30 years	144	37.2	9	42.9	
30–39 years	70	18.1	4	19.0	
40–49 years	52	13.4	4	19.0	
50–59 years	46	11.9	3	14.3	
60–69 years	40	10.3	1	4.8	
≥70 years	35	9.0	0	–	
**Gender**					**<0.001**^S^
Male	387	100.0	0	–	
Female	0	–	21	100.0	
**Hernia side**					0.3 ^NS^
Right	199	51.4	14	66.7	
Left	176	45.5	7	33.3	
Bilateral	12	3.1	0	–	
**Hernia type**					**0.03**^S^
Direct	85	22.0	0	–	
Indirect	294	76.0	21	100.0	
Direct and indirect	8	2.1	0	–	
**Previous recurrence**					0.1 ^NS^
Yes	40	10.3	0	–	
No	347	89.7	21	100.0	
**Follow up duration**					0.3 ^NS^
Five years	150	38.8	6	28.6	
Ten years	237	61.2	15	71.4	

S=Significant, NS=Not significant.

No significant differences were observed between inguinal hernia patients with postoperative complications and inguinal hernia patients without postoperative complications regarding smoking (p = 0.07), diabetes mellitus (p = 0.5), obesity (p = 0.4), chronic lung disease (p = 0.7), chronic bronchitis (p = 0.2), chronic cough (p = 0.6) and immunosuppressant (p = 0.09). There was a highly significant association between anemic inguinal hernia patients and post Modified Halsted operation complications (p < 0.001). A highly significant association was observed between collagen disease of inguinal hernia patients and post Modified Halsted operation complications (p < 0.001) (Table 5).

**Table 5:** Distribution of inguinal hernia risk factors according to postoperative complications.

Variable	Complications				P
	Yes		No		
	**No.**	**%**	**No.**	**%**	
**Smoker**					0.07 ^NS^
Yes	127	32.8	3	14.3	
No	260	67.2	18	85.7	
**DM**					0.5 ^NS^
Yes	77	19.9	3	14.3	
No	310	80.1	18	85.7	
**Obese**					0.4 ^NS^
Yes	39	10.1	1	4.8	
No	348	89.9	20	95.2	
**Anemia**					**<0.001**^S^
Yes	19	4.9	5	23.8	
No	368	95.1	16	76.2	
**Chronic lung disease**					0.7 ^NS^
Yes	25	6.5	1	4.8	
No	362	93.5	20	95.2	
**Chronic bronchitis**					0.2 ^NS^
Yes	17	4.4	2	9.5	
No	370	95.6	19	90.5	
**Chronic cough**					0.6 ^NS^
Yes	48	12.4	2	9.5	
No	339	87.6	19	90.5	
**Collagen disease**					**<0.001**^S^
Yes	4	1.0	3	14.3	
No	383	99.0	18	85.7	
**Immunosuppressant**					0.09 ^NS^
Yes	21	5.4	3	14.3	
No	366	94.6	18	85.7	

S=Significant, NS=Not significant.

## 4 Discussion

Success of surgical repair for patients with inguinal hernia is assessed through, low postoperative complication rate, low cost, earlier rehabilitation and no recurrence of hernia. Before advancement of hernia repair, a recurrence rate of 15% was accepted, however, after development of mesh repair and laparoscopy techniques, the recurrence rate was declined [21].

Present study showed no recurrence rate for patients with inguinal hernia after surgical repair using modified Halsted's method during follow up duration between 5 and 10 years. Our study finding is close to results of Maneck et al. [22] retrospective study in Germany, which showed a recurrence rate of (0.95%). The recurrence rate in our study is better than rate of (10%) reported by Liem et al. [23] study in Netherlands for conventional surgical repair of inguinal hernia after 4 years follow up. Our study finding regarding recurrence rate is better than results of Assakran et al. [24] single center retrospective study in Saudi Arabia on 64 patients with inguinal hernia treated by laparoscopy repair which reported that recurrence of inguinal hernia was observed in (6.3%) of patients after four years follow up. Recurrence rates of inguinal hernia following primary surgical repair are less than 1% in American specialized centers to 30% in general population [21]. The recurrence rate of inguinal hernia is different according to surgical method or technique applied as the Shouldice surgical repair is accompanied with lower recurrence rate as compared to other techniques (Bassini, modified Halsted's) [21] and the laparoscopy approach for inguinal hernia repair is associated with lower recurrence rate as compared to open surgery [25]. No recurrence of inguinal hernia in present study might be attributed to high experience of surgeon with inguinal hernia, better modified Halsted's technique steps used by strengthening the posterior wall of the inguinal canal with polypropylene mesh, and obliteration of the inguinal canal by doing double breast suturing of the EOA. Although excellent findings of current study, the follow up duration was not too long enough as Köckerling et al. [26] stated that inguinal hernia recurrence needs ascertainment after 50 years follow up.

The current study found that postoperative complications for inguinal hernia repair with modified Halsted's technique was observed in 94.9% of hernia patients. This rate of complications is higher than complications rate detected by Andersen et al. [27] study in Denmark which reported that overall complications rate of inguinal hernia repair by open surgery was (13%). Our study finding regarding complications is also higher than results of Khalaf prospective clinical study in Iraq which included 250 patients with inguinal hernia and revealed that 12% of them developed postoperative complications [28]. This higher rate of complications is due to involvement of oedema of the cord in the complications. Oedema of the cord represented 97.4% of the reported complications in current study and it might be attributed to tightening of the cord, this oedema is transient and in most cases disappeared within few days. Other complications were pain on exertion, wound seroma and wound hematoma. These findings are in agreement with results of recent retrospective study conducted by Chibata and Daronch in Brazil on records of 313 patients with inguinal hernia [29].

In present study, there was a highly significant association between male gender hernia patients and postoperative complications (p < 0.001). This finding is similar to results of Köckerling et al. [30] study in Germany, which referred to gender differences in severity of inguinal hernia and rate of postoperative complications. Our study showed a highly significant association between direct hernia type and postoperative complications (p < 0.001). This finding is similar to results of Patil et al. [31] study in India. The significant patients' risk factors related to postoperative complications following inguinal hernia repair in current study were anemia and collagen disease. This finding coincides with results of Weyhe et al. [32] study in Germany and Harrison et al. [33] study in USA.

Main study limitations were the cross sectional design limitation of temporal relationship difficulty, missing or lost data and single center study.

This study concluded that modified Halsted's operation for inguinal hernia repair is effective in treatment of inguinal hernia with low recurrence and complications rate. The common postoperative complication of modified Halsted's operation is oedema of the cord. Risk factors related to postoperative complications are male gender, direct inguinal hernia type, anemia and collagen diseases. This study recommends use of new modified Halsted's technique in treating inguinal hernia and well training of surgeons to this new technique to avoid recurrence and complications especially in countries with low resources and poor health infrastructure.

## Please state any conflicts of interest

None.

## Please state any sources of funding for your research

I declared all sources of funding. And declare the role of study sponsors, if any, in the collection, analysis and interpretation of data; in the writing of the manuscript; and in the decision to submit the manuscript for publication.

## Consent

Written informed consent was obtained from the patient for publication.

Informed consent paper:

Dear patients: This study aimed to assess Modified Halsted's operation for inguinal hernia repair.

This study obtained information directly from you and other data during the surgical operation.

Your participation in study as volunteer and not mandatory or financed.

You have the right to not participate in this study and this will not affect the quality of the surgical operation.

You have the right to withdrawal from the study at any time of research.

The researcher respects your confidentiality.

Researcher.

## Registration of research studies

1 Name of the registry: www.researchregistry.com.
2 Unique Identifying number or registration ID: 7177.
3 Hyperlink to registration:

https://www.researchregistry.com/browse-the-registry#home.

(Researchregistry 7177. Available at: www.researchregistry.com

www.researchregistry.com/browse-the-registry#home).

## Guarantor

No Guarantor.

## Ethical approval

- Informed written consent was obtained after explaining the nature of the operation and its risks.
- The approval of ethics committee was obtained from Health Ethics Committee in college of medicine, university of Baghdad with a registration number: 149.
- Ethical considerations were obtained according to Helsinki Declaration.

## Funding

No funding.

## Author contribution

Single author, did the study concept or design, data collection, data analysis or interpretation, writing the paper.

## Declaration of competing interest

Declared none.

## Provenance and peer review

Not commissioned, externally peer-reviewed.
